# Rhinacanthin C Inhibits Osteoclast Differentiation and Bone Resorption: Roles of TRAF6/TAK1/MAPKs/NF-κB/NFATc1 Signaling

**DOI:** 10.1371/journal.pone.0130174

**Published:** 2015-06-17

**Authors:** Mineko Tomomura, Ryuichiro Suzuki, Yoshiaki Shirataki, Hiroshi Sakagami, Nobuaki Tamura, Akito Tomomura

**Affiliations:** 1 Meikai Pharmaco-Medical Laboratory (MPL), Meikai University School of Dentistry, Sakado, Saitama, Japan; 2 Division of Biochemistry, Department of Oral Biology and Tissue Engineering, Meikai University School of Dentistry, Sakado, Saitama, Japan; 3 Laboratory of Pharmacognosy and Natural Medicines, Faculty of Pharmaceutical Sciences, Josai University, Sakado, Saitama, Japan; 4 Division of Pharmacology, Department of Diagnostic and Therapeutic Sciences, Meikai University School of Dentistry, Sakado, Saitama, Japan; 5 First Division of Oral and Maxillofacial Surgery, Department of Diagnostic and Therapeutic Sciences, Meikai University School of Dentistry, Sakado, Saitama, Japan; Université de Lyon - Université Jean Monnet, FRANCE

## Abstract

Rhinacanthin C is a naphthoquinone ester with anti-inflammatory activity, found in *Rhinacanthus nasutus* (L) Kurz (Acanthaceae). We found that rhinacanthin C inhibited osteoclast differentiation stimulated by the receptor activator of nuclear factor-κB ligand (RANKL) in mouse bone marrow macrophage cultures, although the precise molecular mechanisms underlying this phenomenon are unclear. In this study, we investigated the inhibitory mechanisms of rhinacanthin C in osteoclastogenesis. Rhinacanthin C suppressed RANKL-induced nuclear factor of activated T cells c1 (NFATc1) expression. Phosphorylation of ERK, JNK, and NF-κB, but not p38, was inhibited by rhinacanthin C, which also inhibited RANKL-stimulated TRAF6-TAK1 complex formation. Thus, the anti-osteoclastogenic effect of rhinacanthin C is mediated by a cascade of inhibition of RANKL-induced TRAF6-TAK1 association followed by activation of MAPKs/NF-κB; this leads to suppression of c-Fos and NFATc1, which regulate transcription of genes associated with osteoclast differentiation. In vivo, rhinacanthin C also reduced RANKL-induced osteoclast formation and bone resorption in mouse calvaria. Rhinacanthin C also suppressed LPS-stimulated osteoclastogenesis and bone resorption in vitro and in vivo. Rhinacanthin C may provide a novel therapy for abnormal bone lysis that occurs during inflammatory bone resorption.

## Introduction

Bone formation by osteoblasts and bone resorption by osteoclasts are balanced to maintain bone homeostasis. Enhanced bone resorption can lead to impaired bone structure and bone fracture in osteoporosis, rheumatoid arthritis, and bone metastatic disease.

Osteoclasts differentiate from monocyte/macrophage lineage precursor cells; they are multinucleated giant cells with bone-resorbing activity. Receptor activator of nuclear factor-κB ligand (RANKL), proinflammatory cytokines (TNF-α, IL-1), and lipopolysaccharide (LPS) induce osteoclast differentiation and activation [1–4]. RANKL, which is produced by osteoblasts and bone marrow stromal cells, binds to RANK on monocyte/macrophages followed by receptor oligomerization and recruitment of signaling adapter molecules such as TNF receptor-associated factor-κB ligand 6 (TRAF6). Subsequent TRAF6-TAK1 association activates the downstream signaling pathways NF-κB and activator protein 1 (AP-1) (c-Fos/c-Jun dimer) and induces expression of NFATc1, which is the master osteoclast regulator [1,5]. Interference with these signaling pathways may prevent excessive osteoclast formation and pathological bone loss.

Herbal extracts are widely used as traditional medicines. Screening for phytochemicals with anti-osteoclastogenic activity may lead to the discovery and development of novel drugs to protect against or treat osteopenia and osteoporosis with minimal side effects. The shrub *Rhinacanthus nasutus* (L) Kurz (Acanthaceae) is distributed in Southeast Asia and is used in traditional medicines for the treatment of pneumonia, diabetes, hypertension, and skin diseases. The root and aerial portions of *R*. *nasutus* contain naphthoquinone esters, such as rhinacanthins C, D, N, and Q, which exhibit anti-inflammatory, anti-allergic, anti-tumor, and anti-viral activities [6–10]. Recently, we reported anti-osteoclastogenic activity in the ethyl acetate-soluble fraction of a methanol extract of the *R*. *nasutus* root [11]. We isolated five components (rhinacanthins C, G, N, and Q, and rhinacanthone) from the ethyl acetate-soluble fraction of this plant and found rhinacanthin C is a potent inhibitor of RANKL-stimulated osteoclastogenesis in mouse bone marrow macrophage (BMM) cultures [12]. In this study, we investigated the molecular mechanism by which rhinacanthin C suppresses RANKL-induced osteoclastogenesis of BMMs and validated its efficacy in vivo.

## Materials and Methods

### Materials

Rhinacanthin C was isolated from the root of *R*. *nasutus* [12]. The structure and purity of rhinacanthin C isolated from *R*. *nasutus* were confirmed by nuclear magnetic resonance (NMR) spectroscopy. No impurities were detected on the ^1^H NMR and ^13^C NMR spectra of rhinacanthin C (S1 Fig). Rhinacanthin C was dissolved in dimethyl sulfoxide (DMSO; Sigma-Aldrich, St. Louis, MO), the final concentration of which in culture media was less than 0.2% (v/v). Antibodies against ERK, phospho-ERK (Thr202/Tyr204), JNK, phospho-JNK (Thr183/Tyr185), IκB-α, phospho-IκB-α (Ser32), NF-κB p65, phospho-NF-κB p65 (Ser536), integrin β3, and c-Src were obtained from Cell Signaling Technology (Danvers, MA). Anti-TRAF6 and anti-NFATc1 antibodies were purchased from Santa Cruz Biotechnology (Santa Cruz, CA). Anti-TAK1 and anti-c-Fos antibodies were purchased from Sigma-Aldrich. An antibody against β-actin was purchased from Medical and Biological Laboratories (Nagoya, Japan). Secondary antibodies were purchased from Santa Cruz and Pierce (Rockford, IL). Alexa Fluor 488-conjugated zymosan A (*Saccharomyces cerevisiae*) BioParticles were purchased from Life Technologies (Carlsbad, CA). LPS from *Escherichia coli* O55:B6 was purchased from Sigma-Aldrich. Recombinant human soluble RANKL was purchased from Oriental Yeast (Tokyo, Japan). Male C57BL/6 mice (aged 8- weeks) and ddy mice (aged 8-weeks) were purchased from Sankyo Lab Service (Ibaraki, Japan). OPG knockout mice were provided by Dr. Udagawa of the Institute for Oral Science at Matsumoto Dental University

### Macrophage formation and phagocytosis assay

*In vitro* macrophage colony formation in mouse bone marrow cells was measured according to manufacturer instructions (Stemcell Technologies, Vancouver, Canada). Briefly, bone marrow cells (BMCs: 1 × 10^4^ cells/mL) were cultured for 7 days in MethoCult (methylcellulose) in the presence of macrophage colony-stimulating factor (M-CSF, 10 ng/mL; R & D Systems, Minneapolis, MN) alone or with rhinacanthin C. Macrophage colonies were counted by phase-contrast microscopy. For the macrophage phagocytosis assay, bone marrow macrophages (BMMs) were cultured in 96-well plates for 3 days in the presence of M-CSF with or without rhinacanthin C. Fluorescein-conjugated zymosan A BioParticles (5 μg) were added and incubated for 30 min. After washing, cell-incorporated zymosan particles were visualized and counted by fluorescence microscopy.

### Osteoclast formation from bone marrow cells

Osteoclasts were differentiated from bone marrow macrophages as described [13]. Briefly, BMCs collected from the femurs and tibias of 8 week-old ddY mice were cultured in α-MEM containing 10% FBS and macrophage colony-stimulating factor (M-CSF, 10 ng/mL; R & D Systems, Minneapolis, MN) for 3 days. The bone marrow macrophages were cultured in M-CSF alone or M-CSF plus RANKL (10 ng/mL; R & D Systems) with or without rhinacanthin C for 3 days. In experiments testing the effects of rhinacanthin C on the LPS-induced osteoclastogenesis, BMMs were primed with RANKL(1 ng/mL) for 24 h before LPS treatment.

### Osteoclast differentiation and bone resorption activity

Tartrate-resistant acid phosphatase (TRAP) activity, an osteoclast differentiation marker, was measured as described [13]. Histochemical staining of TRAP was performed using a leukocyte acid phosphatase kit (Sigma-Aldrich). Cultured cells were fixed in 100% methanol for 1 min at room temperature and air-dried. After TRAP staining, TRAP-positive multinucleated (>3 nuclei) cells (MNCs) were imaged by phase-contrast microscopy. Bone resorption activity was assessed by resorption pit formation on dentin slices as described [14]. Briefly, BMMs cultured on dentin slices with M-CSF plus RANKL in the presence or absence of rhinacanthin C for 6 days were scraped off and the resorption pits were stained with acid-hematoxylin solution (Sigma-Aldrich). The absorbed pit areas were quantified with NSI Element imaging software (Nikon Instech, Tokyo, Japan).

### Western blotting and immunoprecipitation

For western blotting analysis, whole-cell lysates were prepared with SDS sampling buffer or RIPA I buffer (25 mM HEPES, pH 7.7, 0.3 M NaCl, 1.5 mM MgCl_2_, 0.2 mM EDTA, 0.1% Triton X-100, 10 mM β-glycerophosphate, 1 mM NaF, 1 mM Na_3_VO_4_) supplemented with protease inhibitor cocktail (Roche Applied Science, Basel, Switzerland). Samples were separated by SDS-PAGE, transferred onto Immobilon-P PVDF membranes (Millipore, Billerica, MA), and then immunoblotted with primary antibody. The blots were incubated with horseradish peroxidase-conjugated secondary antibodies for 1 h, and chemiluminescence was detected with an Immobilon system (Millipore). For immunoprecipitation, cell lysates extracted with RIPA I buffer were incubated with anti-TRAF6 or anti-TAK1 antibody for 2 h at 4°C, followed by 1-h incubation at 4°C with protein G-Sepharose beads (GE Healthcare Bio-Sciences, Piscataway, NJ). After washing with RIPA II (20 mM HEPES, pH 7.7, 0.15 M NaCl, 2.0 mM MgCl_2_, 0.15 mM EDTA, 0.05% Triton X-100) buffer, immune complexes were analyzed by western blotting. Protein band intensities were quantified using ImageJ.

### Cell viability

Viability was evaluated using a cell proliferation assay kit (Promega, Madison, WI). At the end of culture, a 1/10 volume of reagent was added and incubated for 2 h. Absorbance was measured at 492 nm.

### RANKL and endotoxin-induced bone loss *in vivo*

Eight-week-old male mice (ddy) were divided into three groups (n = 3–4/group). Vehicle (30% DMSO) or RANKL (0.7 mg/kg body weight) with or without rhinacanthin C (2 mg/kg body weight) was injected daily into the subcutaneous tissue overlying the calvaria under anesthesia with pentobarbital (Somnopentyl, Kyoritsu Seiyaku; 40–50 mg/kg body weight). Eight-week-old normal (wild-type) male or osteoprotegerin (OPG) knockout mice (OPG-/-) (C57BL/6) were used for the LPS experiments. Vehicle (30% DMSO) or lipopolysaccharide (LPS, 3.3 mg/kg body weight; Sigma-Aldrich) with or without rhinacanthin C (2 mg/kg body weight) was injected. The mice were sacrificed on day 5. Calvariae were dissected, fixed in 4% paraformaldehyde, and stained for TRAP activity. Three-dimensional reconstruction images of calvarial bone were obtained by micro-focal computed tomography (μCT) scanning (MCT-CB130MF; Hitachi Medical, Tokyo, Japan) at the Research Institute of Bone Structure Analysis (Yokosuka, Kanagawa, Japan). The X-ray tube was operated at an acceleration voltage of 60 kV and current of 100 μA. The geometry of the setup and resulting constraints in resolution lead to a voxel size of 32 × 32 × 32 μm. The 3-D images were analyzed using Tri-3D Bon software (Ratoc System Engineering, Tokyo, Japan). Trabecular bone volume (BV; mm^3^), total tissue volume (TV; mm^3^), bone volume fraction (BV/TV; %) were calculated in 3D mode. Static bone morphometric parameters trabecular separation (Tb.Sp; μm), and trabecular bone pattern factor (TBPf; 1/mm) were also calculated.

### Ethics Statement

This study was performed according to the Fundamental Guidelines of the Ministry of Education, Culture, Sports, Science and Technology of Japan for Proper Conduct of Animal Experiment and Related Activities in Academic Research Institutions. The Animal Care and Use Committee of Meikai University approved all animal experiments.

All animal experiments were performed under anesthesia to limit pain. The mice were sacrificed by spinal cord dislocation under anesthesia with pentobarbital.

### Statistical analysis

Data were analyzed by ANOVA and are presented as the means ± SD of at least three independent experiments. In all analyses, *P* < 0.05 was taken to indicate statistical significance.

## Results

### Rhinacanthin C inhibits RANKL-mediated osteoclast formation from mouse BMMs without cytotoxicity

**Fig 1:**
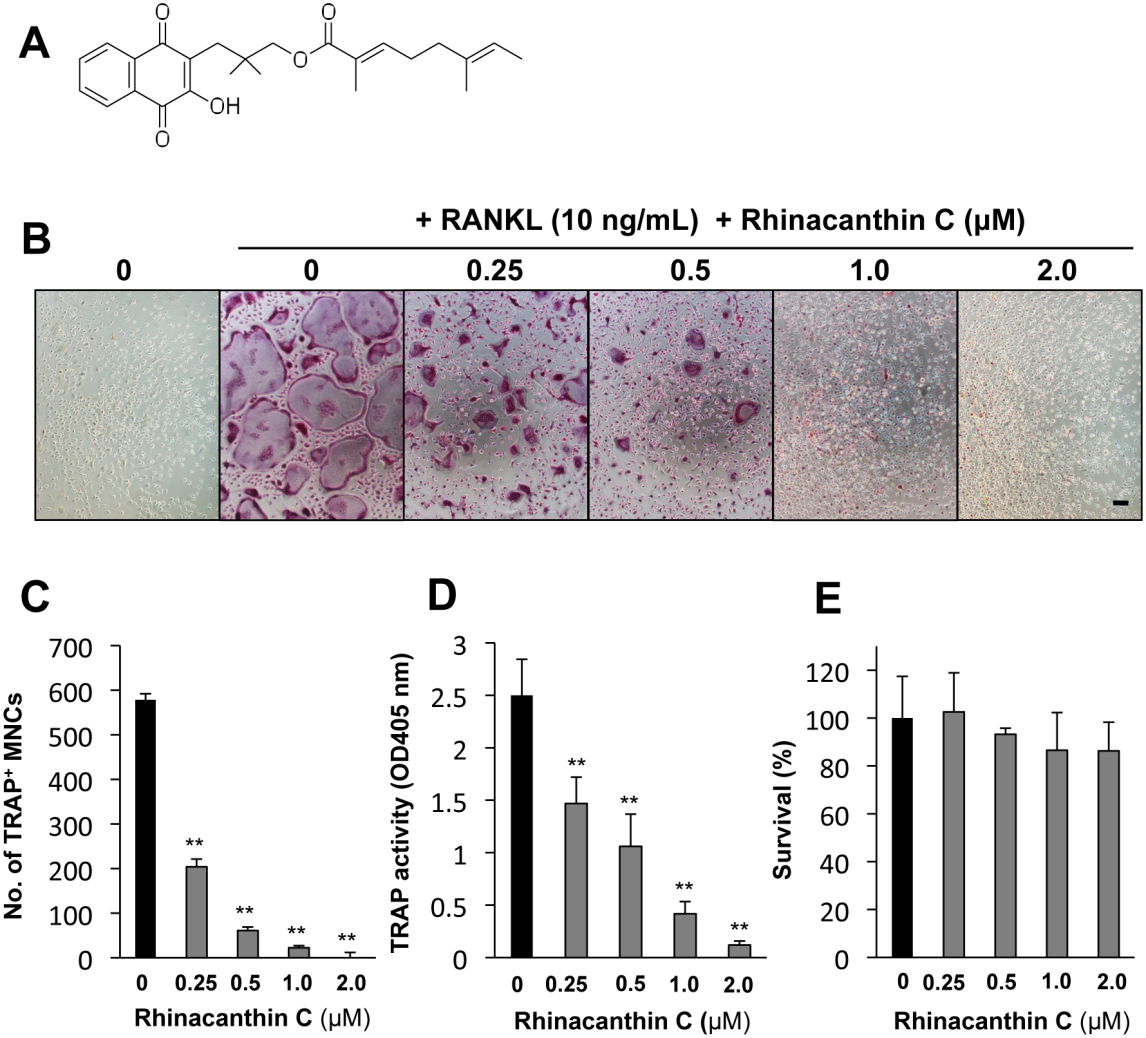
Effects of rhinacanthin C on osteoclastogenesis and cell viability in BMM cultures. **A,** Chemical structure of rhinacanthin C. **B,** BMCs were cultured for 3 days with M-CSF (10 ng/mL), then with M-CSF alone or M-CSF plus RANKL (10 ng/mL) in the presence or absence of rhinacanthin C (0.25–2.0 μM). The cells were stained for TRAP activity. Bar, 100 μm. **C**, Dose-dependent effects of rhinacanthin C on RANKL-induced TRAP-positive multi-nuclear (>3 nuclei) cell (MNC) formation from BMCs. **D,** Dose-dependent rhinacanthin C inhibition of TRAP activity in the medium of BMM cultures in the presence of RANKL and M-CSF. **E,** Viability was determined by MTT assay after 3 days. Data are expressed as means ± SD of three experiments. **P < 0.01 vs. untreated controls.

Rhinacanthin C is a naphthoquinone derivative containing an alkyl side chain (Fig 1A). We previously reported that rhinacanthin C is a strong inhibitor of RANKL-stimulated TRAP-positive multinucleated cell formation from mouse BMMs [12]. In this study, we investigated the effects of rhinacanthin C on osteoclast differentiation and bone resorption pit formation. Rhinacanthin C produced dose-dependent inhibition of RANKL-induced TRAP-positive multinuclear osteoclast formation and TRAP activity in BMM culture (0.25–2.0 μM) (Fig 1B to 1D). In our previous study, rhinacanthin C exhibited non-apoptotic cytotoxicity against tumor cells [12]. To investigate whether osteoclastogenesis inhibition by rhinacanthin C is due to its cytotoxicity, we measured the viability of RANKL-induced osteoclasts exposed to rhinacanthin C. Fig 1E shows that rhinacanthin C did not suppress viability even at 2.0 μM, at which concentration it abolished RANKL-stimulated osteoclast formation. Thus, the inhibitory effects of rhinacanthin C on osteoclastogenesis seemed not to be due to cytotoxicity.

Osteoclasts differentiated from the monocyte-macrophage lineage. Therefore, we examined the effects of rhinacanthin C on macrophage colony formation with M-CSF from BMCs. The macrophage-type colonies formed from BMCs did not differ in the absence or presence of rhinacanthin C (S2A Fig). Furthermore, the numbers of zymosan-incorporated phagocytic macrophages did not significantly differ in the presence of absence of rhinacanthin C (S2B Fig). Thus, macrophage formation and phagocytic function stimulated by M-CSF are not inhibited by rhinacanthin C. We further characterized the effects of rhinacanthin C on osteoclastogenesis by performing two experiments. First, BMCs were cultured with rhinacanthin C for 3 days in the presence of M-CSF, followed by RANKL plus M-CSF to induce osteoclast formation. In the second experiment, BMCs were cultured with rhinacanthin C and then the BMMs were cultured with RANKL plus M-CSF, also in the presence of rhinacanthin C. In experiment 1, RANKL-stimulated osteoclast formation remained essentially the same, regardless of pretreatment with rhinacanthin C. However, after RANKL stimulation, rhinacanthin C provided dose-dependent inhibition of osteoclast formation, as shown in experiment 2 (S2D Fig). These results suggest the inhibitory role of rhinacanthin C in osteoclastogenesis does not occur at the stage macrophage formation and function as an osteoclast progenitor but at the stage of RANKL-stimulated osteoclast formation step.

Next, we sought to identify what stage of osteoclast differentiation is more strongly affected by rhinacanthin C. Rhinacanthin C was added at various stages of osteoclastogenesis over 3 days (S3A Fig). Late addition of rhinacanthin C (3^rd^ day, i.e., 2+1RC) had little effect on TRAP activity (S3C Fig) and TRAP positive-multinucleated cell formation (S3B and S3D Fig). Treatment on days 1 and 2 (2RC+1) resulted in a significantly lower number of TRAP positive-multinucleated cells versus treatment on days 2 and 3 (1+2RC), indicating that rhinacanthin C suppressed early-to-middle stage RANKL-stimulated osteoclastogenesis form BMMs. The numbers of TRAP-positive multinucleated cells and TRAP activity were higher in cultures treated on day 1 (1RC+2) than in those treated on day 2 (1+1RC+1). These results suggest the inhibitory effects of rhinacanthin C is reversible, and differentiation potential of BMMs treated with rhinacanthin C on day 1 is recovered over 2 days culture without rhinacanthin C. Indeed, we observed greater numbers of TRAP-positive multinucleated cells in the 1+1RC+1 culture when normal culture conditions were continued for one more day (data not shown). Taken together, these results suggest rhinacanthin C reversibly suppresses the early to middle stage of osteoclast differentiation.

**Fig 2:**
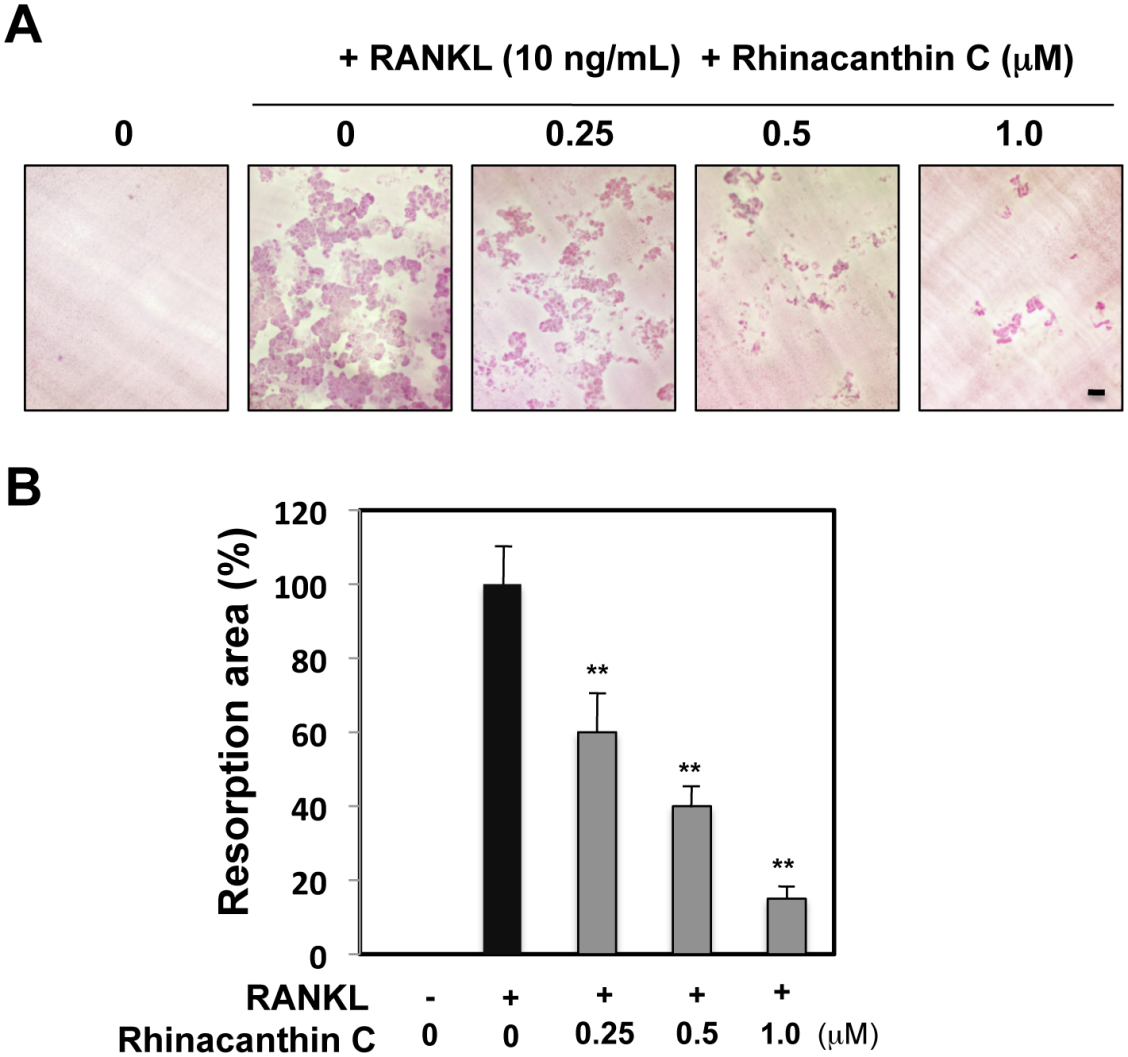
Effects of rhinacanthin C on bone resorption in BMM cultures. **A,** BMCs were cultured on dentin slices for 3 days with M-CSF (10 ng/mL), then for 6 days in the presence or absence of rhinacanthin C in the presence of RANKL (10 ng/mL). After removal of the cells, areas of resorption pits were stained with hematoxylin. Bar, 100 μm. **B,** total resorption pit area was measured and the results are shown as % of RANKL treatment. Data are expressed as means ± SD of three experiments. **P < 0.01 vs. untreated control.

We next examined the effects of rhinacanthin C on bone resorption activity, which reflects the acquisition of differentiated osteoclast function. BMMs were cultured on dentin slices with M-CSF and RANKL for 6 days in the presence or absence of increasing concentrations of rhinacanthin C, and the osteoclastic bone resorption pits were examined. As shown in Fig 2, rhinacanthin C inhibited RANKL-stimulated bone resorption in a dose-dependent manner. These results were consistent with the inhibition of osteoclast differentiation by rhinacanthin C.

### Rhinacanthin C suppresses induction of RANKL-induced proteins involved in osteoclast differentiation and function

**Fig 3:**
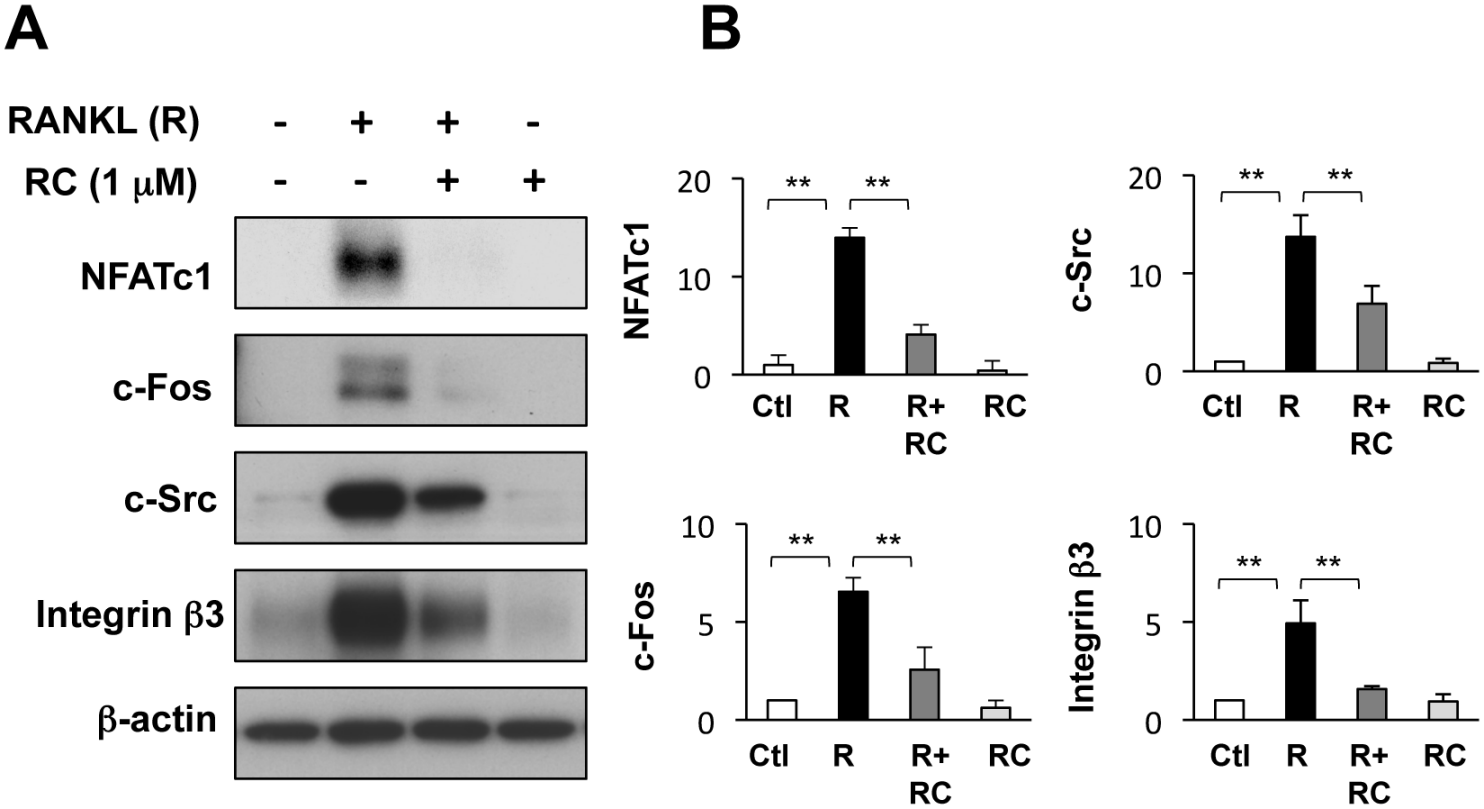
Effects of rhinacanthin C on induction of osteoclast-related proteins by RANKL. **A,** BMCs were cultured for 3 days with M-CSF (10 ng/mL), then for 2 days in the presence or absence of rhinacanthin C (RC, 1 μM) and RANKL (R, 10 ng/mL). Whole-cell extracts were analyzed by western blotting with primary antibodies raised against NFATc1, c-Fos, c-Src, and integrin β3. β-Actin was used as an internal control. **B**, Proteins were normalized to β-actin and expressed as the fold change relative to the untreated control (Ctl). Values are the means ± SD of three to four independent experiments. **P < 0.01.

NFATc1 is a master transcription factor in osteoclastogenesis. c-Fos, a major component of transcription factor AP-1, induces NFATc1 expression to regulate osteoclast differentiation [5,15]. RANKL significantly increased NFATc1 and c-Fos protein levels after 2 days, whereas rhinacanthin C suppressed the RANKL-stimulated induction of both transcription factors (Fig 3). Integrin β3 and c-Src are important for actin ring formation, which is essential for bone resorption [16]. RANKL increased c-Src and integrin β3 expression, while rhinacanthin C suppressed these effects (Fig 3).

### Rhinacanthin C suppresses RANKL-induced activation of NF-κB and MAPKs

**Fig 4:**
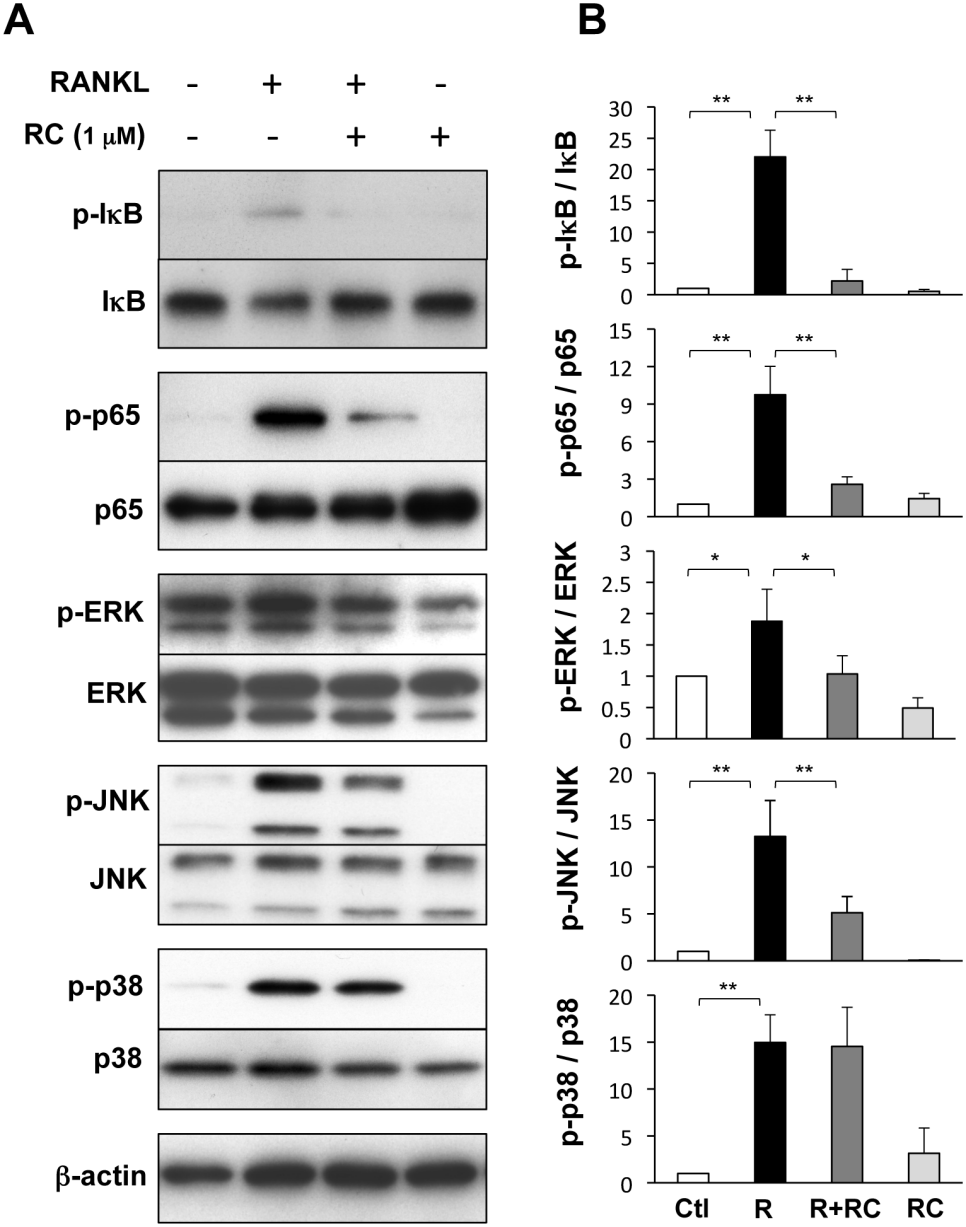
Effects of rhinacanthin C on RANKL-stimulated signaling in BMM. **A,** BMCs were cultured for 3 days with M-CSF (10 ng/mL), then with RANKL (R, 10 ng/mL) for 10 min in the presence or absence of rhinacanthin C (RC, 1 μM). Whole-cell extracts were analyzed by western blotting. **B,** The levels of phosphorylated proteins were quantified, normalized to the total levels of corresponding proteins, and expressed as the fold change vs. the untreated control (Ctl). Values are the means ± SD of three to four independent experiments. *P < 0.05, **P < 0.01.

NF-κB activation is essential for osteoclast differentiation. NF-κB forms a complex with inhibitor IκB, phosphorylation of which leads to degradation and dissociation of IκB from the NF-κB/p65 subunit. Then, phospho-NF-κB/p65 translocates to the nucleus and binds to the promoter of the NFATc1 gene [17]. The MAPK subfamilies ERK, JNK, and p38 are also essential for osteoclast differentiation [18–20]. NF-κB and MAPKs are activated by NFATc1 and c-Fos-integrated RANKL signaling [5,21]. We next examined the effects of rhinacanthin C on RANKL-driven activation NF-κB and MAPKs (Fig 4). Within 10 min, RANKL induced phosphorylation of IκB, NF-κB/p65, ERK, and JNK in BMM culture; however, rhinacanthin C suppressed this RANKL-induced phosphorylation. Rhinacanthin C did not suppress RANKL-induced phosphorylation of p38. Thus, rhinacanthin C suppresses RANKL-mediated activation of the ERK, JNK, and NF-κB signaling axis in osteoclastogenesis.

### Rhinacanthin C suppresses RANKL-induced TRAF6-TAK1 complex formation

**Fig 5:**
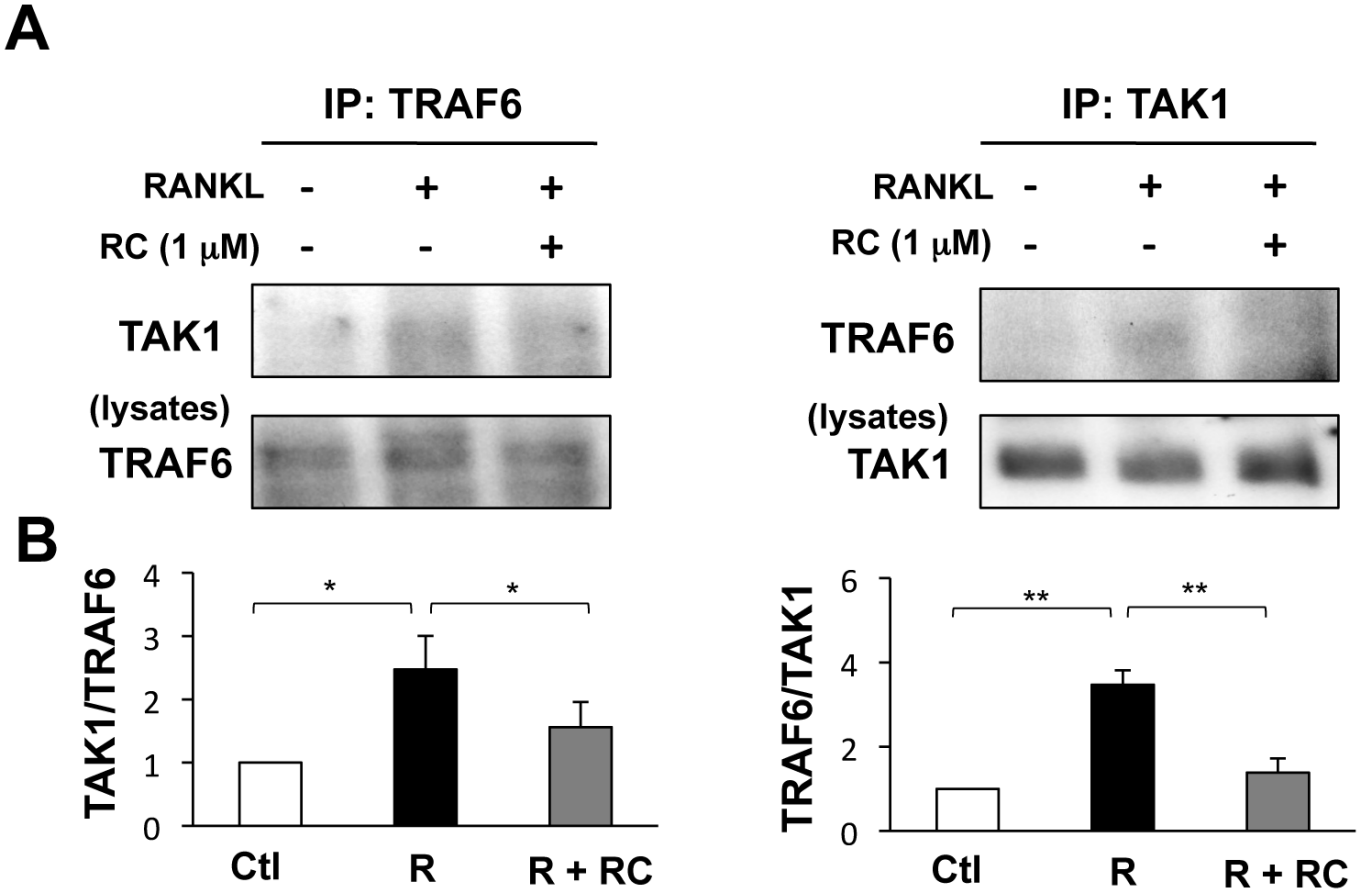
Effects of rhinacanthin C on complex formation of TRAF6 and TAK1. **A,** BMCs were cultured for 3 days with M-CSF (10 ng/mL), then pretreated with rhinacanthin C (RC, 1 μM) or DMSO (0.1%) for 20 min and stimulated with RANKL (R, 10 ng/mL) for 5 min with or without rhinacanthin C. Cell lysates were immunoprecipitated (IP) with anti-TRAF6 or anti-TAK1 and immunoblotted with anti-TAK1 or anti-TRAF6, respectively (upper panel). Expression of TRAF6 and TAK1 in cell lysates was determined by immunoblotting (lower panel). **B,** The level of co-immunoprecipitated TAK1 or TRAF6 was quantified and normalized to total TRAF6 or TAK1, respectively. Data are expressed as the fold change vs. the untreated control. Values are the means ± SD of three independent experiments. *P < 0.05, **P < 0.01.

TRAF6 and TAK1 complex formation is an important step prior to RANKL-mediated MAPK and NF-κB activation in BMMs [22]. As rhinacanthin C suppressed RANKL-stimulated MAPK and NF-κB activation, we next examined whether rhinacanthin C could inhibit TRAF6–TAK1 complex formation. In pull-down assays, co-immunoprecipitation of TAK1 with anti-TRAF6 antibody (and of TRAF6 with anti-TAK1) increased in the presence of RANKL (Fig 5). However, rhinacanthin C suppressed RANKL-induced co-precipitation of TRAF6 and TAK1. Thus, rhinacanthin C inhibits RANKL-stimulated osteoclastogenesis by suppressing TRAF6–TAK1 complex formation, thereby modulating the downstream signaling pathways required for osteoclast-specific gene expression.

### Rhinacanthin C prevents RANKL-induced bone resorption *in vivo*

**Fig 6:**
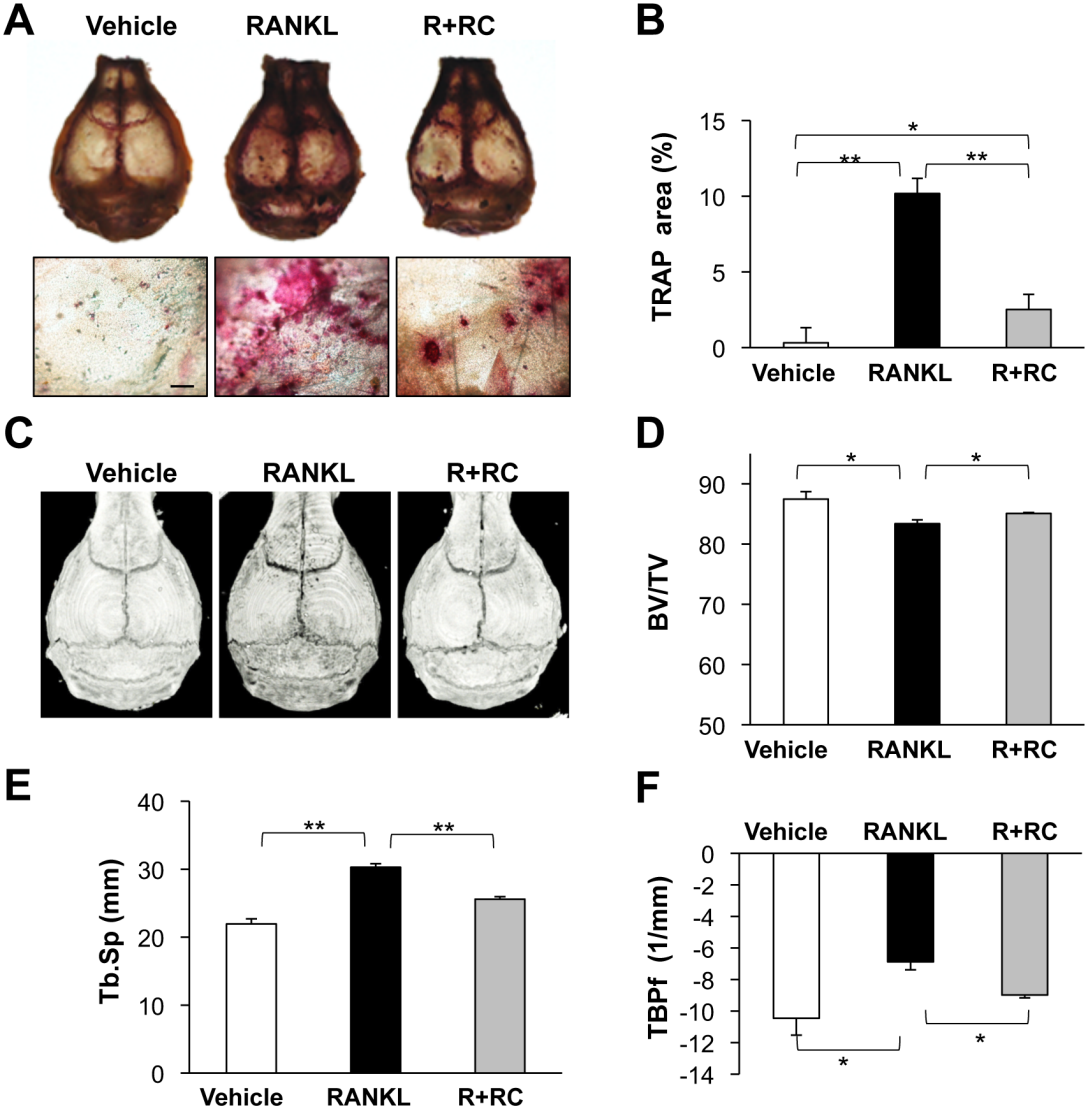
Protective effects of rhinacanthin C on RANKL-induced mouse calvarial osteolysis. Vehicle (30% DMSO) or rhinacanthin C (RC, 2 mg/kg body weight) with or without RANKL (0.7 mg/kg body weight) was daily injected into the subcutaneous tissue overlying the calvaria of 8-week-old ddy mice. The mice were sacrificed on day 5. **A,** TRAP staining of whole calvaria (upper) and high magnification of TRAP stain on calvaria (lower). Bar, 400 μm. **B,** TRAP^+^ area was measured by densitometry using Image J.**C,** Three-dimensional micro-CT image of calvaria. **D,** Bone volume/total tissue volume ratio (BV/TV). **E,** Trabecular separation (Tb.Sp). **F,** Trabecular bone pattern factor (TBPf). *P < 0.05, **P < 0.01.

To determine whether rhinacanthin C prevents RANKL-induced bone loss *in vivo*, RANKL was administered once daily for 5 days with or without rhinacanthin C on the calvaria. We then stained for TRAP activity and performed morphometric analysis of calvarial bone by micro-CT. The TRAP-positive area increased in the RANKL-injected calvaria versus the vehicle-injected control (Fig 6A and 6B). Simultaneous administration of rhinacanthin C reduced the RANKL-induced TRAP-positive area. Representative micro-CT images of RANKL-treated calvaria showed that RANKL treatment increased excavated bone destruction, whereas rhinacanthin C-injected calvaria showed less bone destruction in comparison to RANKL-injection alone (Fig 6C). Micro-CT analysis of RANKL-treated calvaria showed decreased BV/TV, and increased trabecular separation (Tb.Sp) compared with vehicle-treated control calvaria (Fig 6D and 6E). On the other hand, rhinacanthin C treatment significantly increased BV/TV and decreased trabecular separation, suggesting rhinacanthin C prevented RANKL-induced calvarial bone destruction. TBPf is the ratio of convex structures to concave structures on the trabecular surface, reflecting the connectivity of trabecular bone. TBPf was increased in the RANKL-treated group versus the control, suggesting RANKL destroys the microarchitecture of trabecular bone (Fig 6E). Rhinacanthin C reduced the RANKL-induced increase of TBPf. Thus, rhinacanthin C prevents osteoclastic bone resorption *in vitro* and *in vivo*.

### Rhinacanthin C inhibits LPS-induced osteoclastogenesis and bone resorption

**Fig 7:**
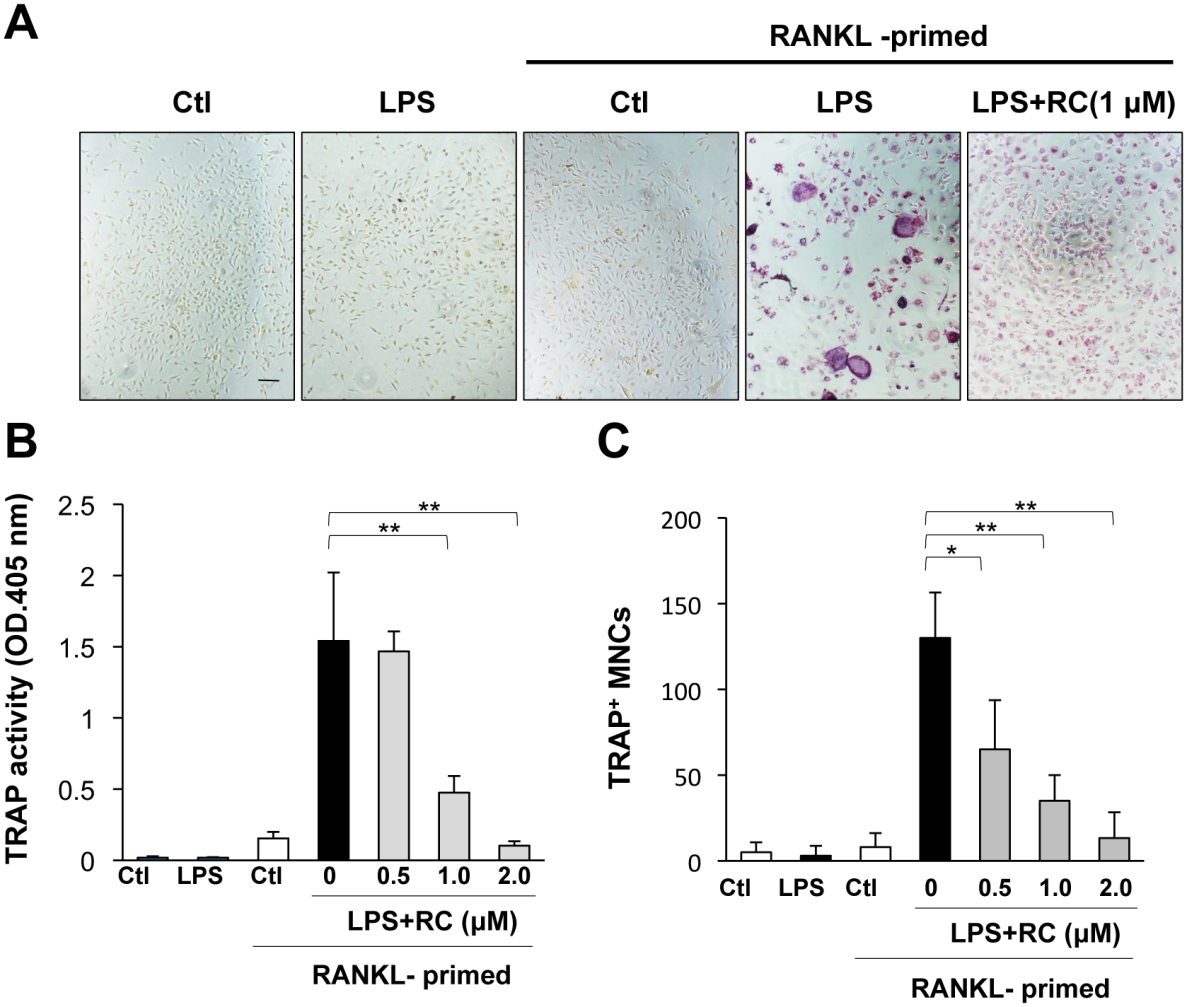
Rhinacanthin C inhibits LPS-induced osteoclastogenesis in RANKL-primed BMMs. **A,** BMMs were primed with RANKL (1 ng/mL) for 24 h, then the culture medium were washed away and the cells were cultured with or without LPS (200 ng/mL) or LPS plus rhinacanthin C (RC, 1 μM). After 3 days, cells were stained with TRAP. Bar, 100 μm. **B,** TRAP activities were measured. **C,** TRAP-positive multinucleated osteoclasts. *P < 0.05, **P < 0.01.

**Fig 8:**
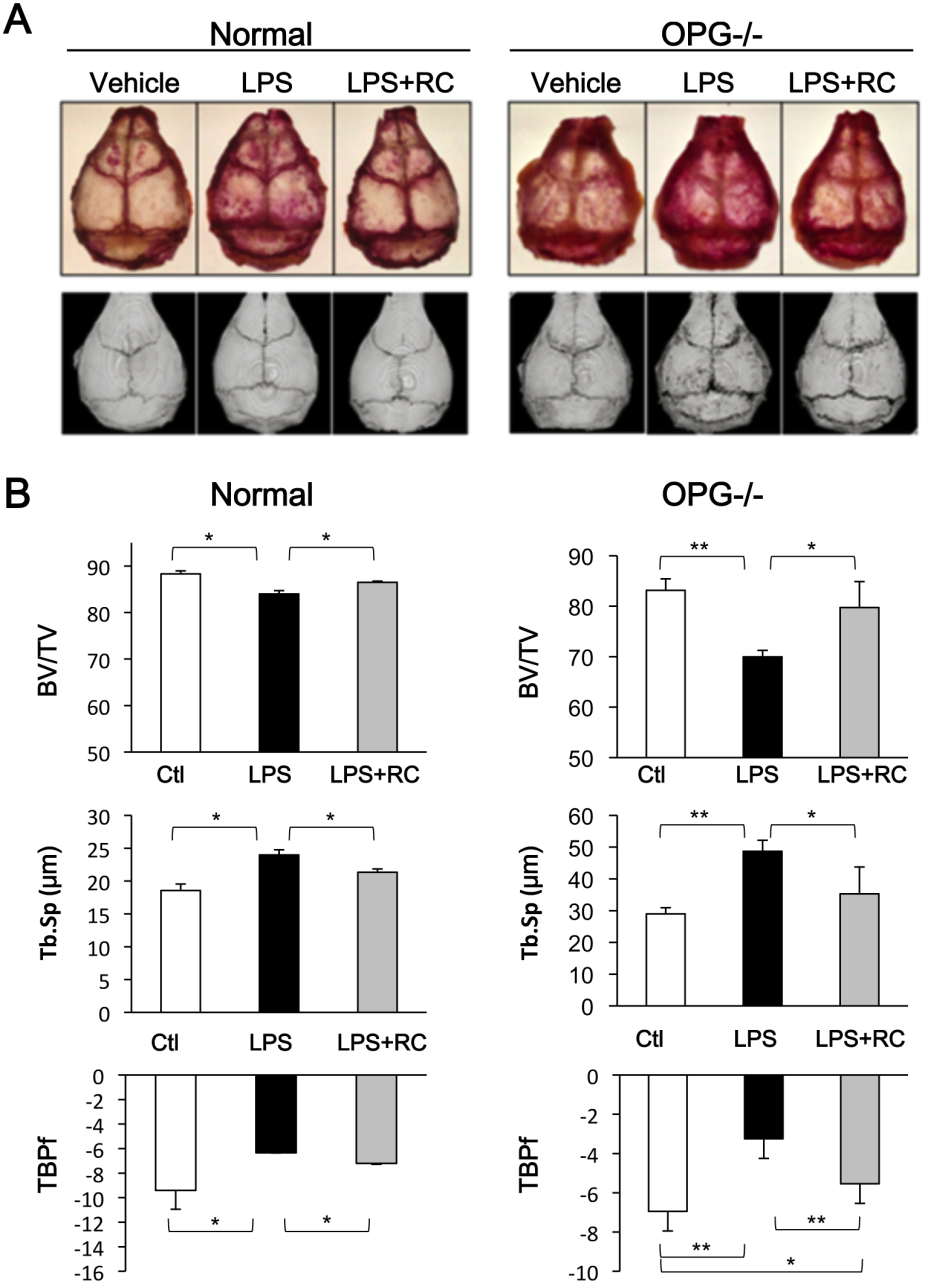
Protective effects of rhinacanthin C on LPS-induced bone destruction *in vivo*. Vehicle (30% DMSO) or LPS (3.3 mg/kg body weight) with or without rhinacanthin C (2 mg/kg body weight) was daily injected into the subcutaneous tissue overlying the calvaria of 8-week-old wild type (normal) and OPG-/- mice. The mice were sacrificed on day 5. **(A)** TRAP staining of whole calvaria and 3D-images. **(B)** Quantitative data of calvarial bone by μCT analysis. BV/TV: Bone volume/total tissue volume ratio. Tb.Sp: Trabecular separation. TBPf: Trabecular bone pattern factor. *P < 0.05, **P < 0.01.

Bacterial infection is associated with bone destruction in periodontitis. LPS, a cell wall component of Gram-negative bacteria, also induces bone resorption [23–25]. We also demonstrated the effects of rhinacanthin C on LPS-induced osteoclastogenesis from BMMs (Fig 7) and bone resorption of mouse calvaria (Fig 8). RANKL priming is needed for LPS-induced osteoclastogenesis [26]. We treated BMMs with RANKL (1 ng/mL) for 24 h followed by LPS treatment with or without rhinacanthin C for 3 days (Fig 7). LPS significantly increased TRAP activity (Fig 7B) and the numbers of MNCs (Fig 7A and 7C) in RANKL-primed BMMs but not in non-primed BMMs. Rhinacanthin C provided dose-dependent inhibition of LPS-stimulated osteoclastogenesis from BMMs.

Finally, we tested the inhibitory effects of rhinacanthin C on LPS-stimulated calvarial bone erosion *in vivo* using normal and OPG-/- mice. Osteoprotegerin-deficient (OPG-/-) mice exhibit osteoporosis due to enhanced osteoclastogenesis caused by a lack of soluble decoy receptor for RANKL [27,28]. As shown in Fig 8, LPS significantly increased TRAP staining of whole calvariae in normal and OPG-/- mice (Fig 8A). Simultaneous administration of rhinacanthin C reduced LPS-induced osteoclast formation. μCT analysis (BV/TV, Tb.Sp, and TBPf) showed that LPS induced bone destruction in OPG-/- mice (Fig 8B). Rhinacanthin C ameliorated LPS-induced calvarial bone resorption of normal and OPG knockout mice. Thus, rhinacanthin C inhibits LPS-induced and RANKL-induced osteoclastogenesis.

## Discussion

Identification and characterization of natural anti-osteoclastogenic compounds from herbal sources support the development of novel therapeutics for patients suffering from abnormal bone lysis and exploration of the regulatory mechanisms underlying osteoclast formation and activation. Previously, we found that rhinacanthin C is a potent inhibitor of RANKL-induced osteoclast formation from BMMs [12]. In this study, we demonstrated the inhibitory mechanisms of rhinacanthin C and its effects in vivo.

Rhinacanthin C suppressed the osteoclast-specific master transcription factor, NFATc1, which regulates osteoclast specific/related gene expression. Indeed, c-Src and integrin β, which are late osteoclastogenesis genes involved in bone resorption, were down-regulated by rhinacanthin C. Therefore, suppression of TRAP activity and pit formation by rhinacanthin C are attributed to reduced levels of osteoclast specific/related gene expression governed by NFATc1. c-Fos induces NFATc1 expression and is an essential regulator of osteoclastogenesis. c-Fos knockout mice develop osteopetrosis due to a lack of osteoclast formation [5,15]. Suppression of NFATc1 accumulation by rhinacanthin C may be caused by down-regulation of c-Fos. The NFATc1 promoter is initially activated by NF-κB and AP-1 in the presence of RANKL [29]. NFATc1 binds to its own promoter, followed by NFATc1-mediated auto-amplification of gene induction [30]. c-Jun and c-Fos (components of AP-1) are phosphorylated and activated by JNK and ERK, respectively [31]. Rhinacanthin C inhibited the RANKL-induced phosphorylation of ERK and JNK. Thus, rhinacanthin C may suppress AP-1 activation *via* inhibition of MAPKs. Rhinacanthin C also suppressed RANKL-induced phosphorylation of IκB and NF-κB/p-65, suggesting that the inhibitory effects of rhinacanthin C on osteoclast differentiation may also be caused by inhibition of NF-κB activity. Thus, rhinacanthin C suppresses the early step of osteoclast differentiation *via* inhibition of RANKL-stimulated NF-κB and MAPK activation.

As a consequence of RANKL binding, RANK activates TRAF6 to initiate complex formation with TAK1, which facilitates NF-κB and MAPK activation [22]. TRAF6 siRNA and TRAF6 decoy peptides inhibit the formation of TRAP-positive multinucleated cell and bone resorption [32]. TRAF6 knockout mice exhibit severe osteopetrosis with defects in bone remodeling caused by impaired osteoclast function [22,33]. Our immunoprecipitation assay revealed that association of TRAF6 and TAK1 was increased by RANKL, while rhinacanthin C suppressed this effect. Rhinacanthin C may disrupt intracellular signal processing by targeting TRAF6–TAK1 complex formation, thus impairing osteoclast differentiation. The direct targets of rhinacanthin C remain unknown. Recently, it was reported that (+)-Vistin A and Norisoboldine inhibit osteoclast differentiation and prevent TRA6-TAK1 formation [34,35]. Whether these natural compounds and rhinacanthin C target the same molecule will be the topic of future research.

Consistent with its anti-osteoclastogenic effects in vitro, rhinacanthin C also inhibits RANKL-induced osteoclast formation and bone destruction *in vivo*. Furthermore, caldecrin inhibits LPS-stimulated osteoclast formation from BMMS and calvarial bone resorption. LPS is recognized by Toll-like receptor (TLR), which triggers recruitment of TRAF6 and TAK1, leading to activation of the NF-κB pathway [36,37]. TRAF6 knockout cells fail to respond to LPS stimulation [38]. Rhinacanthin C might inhibit LPS-induced osteoclastogenesis by suppressing TRAF6-TAK1-NF-κB signaling as it does in RANKL-induced osteoclastogenesis. These results and its reversible effects on osteoclast formation suggest the potential utility of rhinacanthin C in the treatment of conditions associated with abnormal bone lysis such as osteoporosis and inflammation-induced bone loss (rheumatoid arthritis and periodontal bone erosion).

Rhinacanthins are composed of naphthoquinone with variable side chains. Among the rhinacanthins (rhinacanthin C, D, G, N, and Q), rhinacanthin C has the most potent anti-osteoclastogenic activity [12] (data on rhinacanthin D are unpublished). Rhinacanthin C has a 2,6-dimethyl-2,6-octadienoic acid ester acid side chain. The relationship between side chain structure and anti-osteoclastogenic activity are a topic of future research.

In summary, we have demonstrated that rhinacanthin C suppresses RANKL-stimulated TRAF6–TAK1 complex formation and activation of the MAPK/NF-κB/NFATc1 pathways, thereby suppressing expression of osteoclast marker proteins, finally resulting in impaired osteoclast differentiation and activation of bone resorption in cultured BMMs. Rhinacanthin C also suppresses LPS-induced osteoclast formation and bone destruction *in vivo*. The direct targets of rhinacanthin C that mediate its anti-osteogenic activity warrants further study.

## Supporting Information

S1 Fig NMR spectra of rhinacanthin C. **A,** ^1^H NMR spectra of rhinacanthin C. **B,** ^13^C NMR spectra of rhinacanthin C. Spectra were measured on a 400 MHz Agilent-400MR-vnmrs 400 spectrometer (Agilent) in dimethyl sulfoxide-*d*_6_ at room temperature.
(TIF)

S2 Fig Effects of rhinacanthin C on macrophage formation from BMCs. **A**, Macrophage colony formation assays were performed as described in “Materials and Methods.” **B,** Photographs of BMMs after culture in the presence of M-CSF (non). Fluorescein-labeled zymosan particles were incorporated into BMMs. Scale bar, 100 μm. Percentage of zymosan-positive cells to the total after culture in the presence of M-CSF with or without rhinacanthin C (RC). **C** and **D,** TRAP activities in the culture medium. BMCs were cultured in the presence of M-CSF with rhinacanthin C for 3 days, then washed with PBS and exchanged for media without (**C**) or with rhinacanthin C (**D**) in the presence of RANKL. After 3 days, TRAP activities were measured. **P < 0.01
(TIF)

S3 Fig Effects of rhinacanthin C treatment on the stage of RANKL-induced osteoclastogenesis. **A,** Rhinacanthin C (RC, 1 μM) was added to BMM culture at various time points under RANKL stimulation. Black column indicates RC treatment period. Culture medium was exchanged daily. Culture conditions; 3, Continuous treatment without rhinacanthin C for 3 days; 3RC, Continuous treatment with rhinacanthin C for 3 days; 1+2RC, Rhinacanthin C treatment on days 2 to 3; 2+1RC, Rhinacanthin C on day 3; 1RC+2, Rhinacanthin C on day 1; 1+1RC+1, Rhinacanthin C on day 2; 2RC+1, Rhinacanthin C on days 1 to 2. **B**, TRAP staining of osteoclasts cultured by various conditions as described in (A). Bar, 100 μm. TRAP activity (**C**) and number of TRAP-positive multi-nuclear cells (**D**) of osteoclasts are shown. *P < 0.05, **P < 0.01
(TIF)
