# Supplementary figures and images for: Rhinacanthin C Inhibits Osteoclast Differentiation and Bone Resorption: Roles of TRAF6/TAK1/MAPKs/NF-κB/NFATc1 Signaling
Source: PLoS One. 2015 Jun 17;10(6):e0130174. doi: 10.1371/journal.pone.0130174 (PMC4471279; doi:10.1371/journal.pone.0130174)

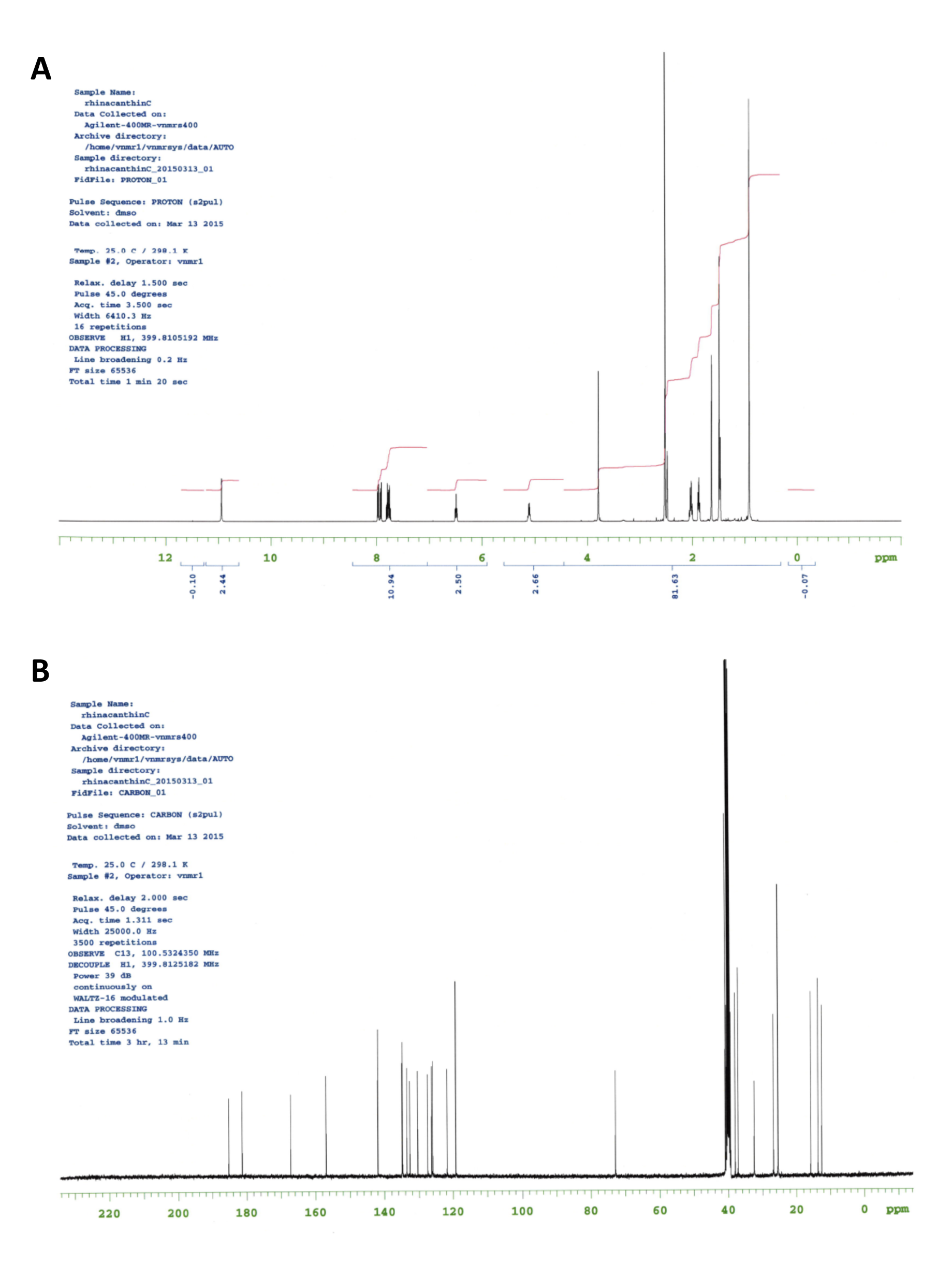

Supplement: S1 Fig — A, 1H NMR spectra of rhinacanthin C. B, 13C NMR spectra of rhinacanthin C. Spectra were measured on a 400 MHz Agilent-400MR-vnmrs 400 spectrometer (Agilent) in dimethyl sulfoxide-d 6 at room temperature. (TIF) [file pone.0130174.s001.tif]

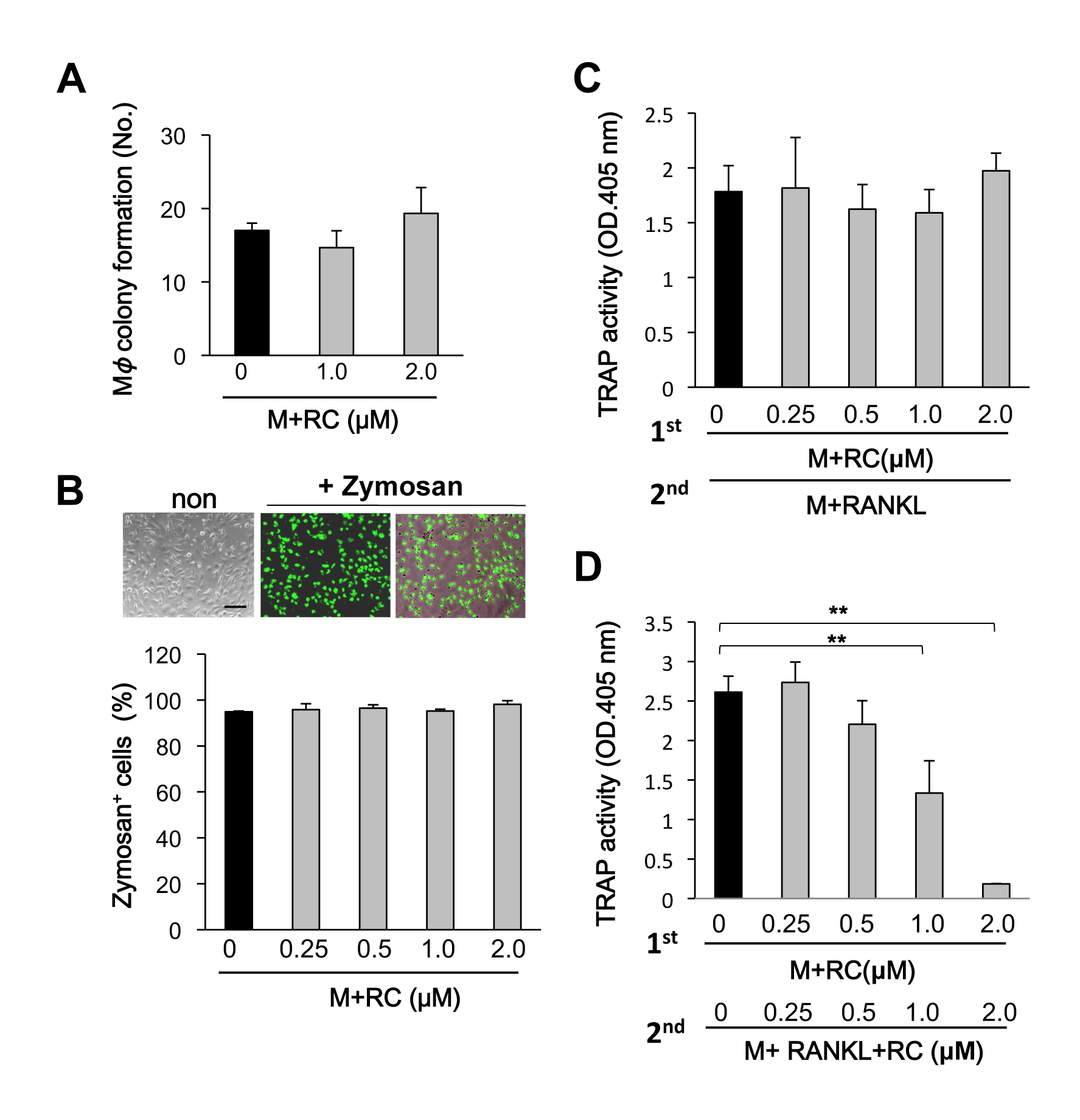

Supplement: S2 Fig — A, Macrophage colony formation assays were performed as described in “Materials and Methods.” B, Photographs of BMMs after culture in the presence of M-CSF (non). Fluorescein-labeled zymosan particles were incorporated into BMMs. Scale bar, 100 μm. Percentage of zymosan-positive cells to the total after culture in the presence of M-CSF with or without rhinacanthin C (RC). C and D, TRAP activities in the culture medium. BMCs were cultured in the presence of M-CSF with rhinacanthin C for 3 days, then washed with PBS and exchanged for media without (C) or with rhinacanthin C (D) in the presence of RANKL. After 3 days, TRAP activities were measured. **P < 0.01 (TIF) [file pone.0130174.s002.tif]

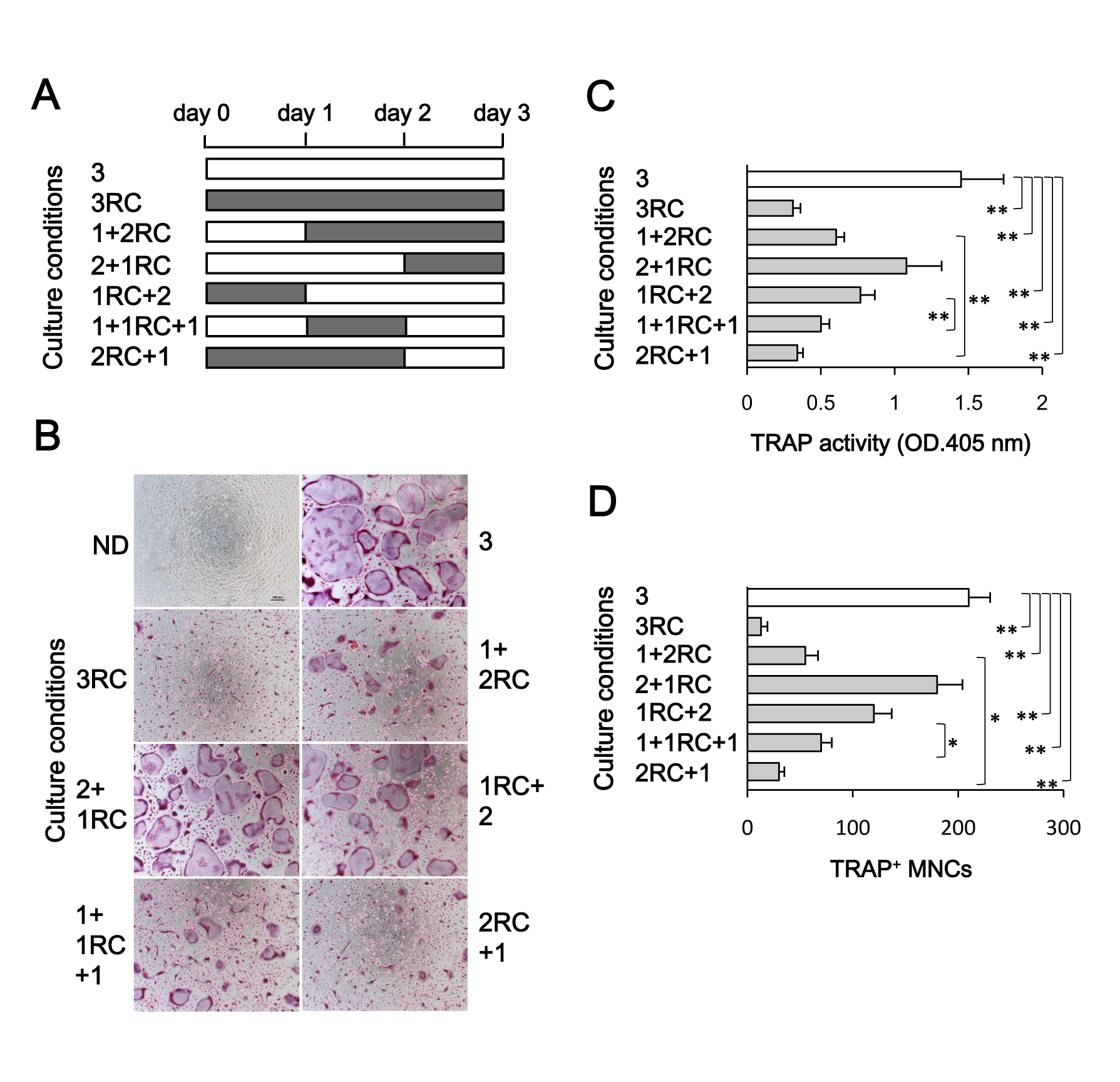

Supplement: S3 Fig — A, Rhinacanthin C (RC, 1 μM) was added to BMM culture at various time points under RANKL stimulation. Black column indicates RC treatment period. Culture medium was exchanged daily. Culture conditions; 3, Continuous treatment without rhinacanthin C for 3 days; 3RC, Continuous treatment with rhinacanthin C for 3 days; 1+2RC, Rhinacanthin C treatment on days 2 to 3; 2+1RC, Rhinacanthin C on day 3; 1RC+2, Rhinacanthin C on day 1; 1+1RC+1, Rhinacanthin C on day 2; 2RC+1, Rhinacanthin C on days 1 to 2. B, TRAP staining of osteoclasts cultured by various conditions as described in (A). Bar, 100 μm. TRAP activity (C) and number of TRAP-positive multi-nuclear cells (D) of osteoclasts are shown. *P < 0.05, **P < 0.01 (TIF) [file pone.0130174.s003.tif]
